# Preparation, Characterization, and pH-Responsive Intestinal Release Properties of Gel Beads Encapsulating Sea Cucumber Mouthpart Peptides

**DOI:** 10.3390/md24080261

**Published:** 2026-07-28

**Authors:** Yige Wu, Lijun Hu, Yue Li, Tiantian Hao, Zhidong Song, Gongming Wang, Chunna Jiao, Jian Zhang

**Affiliations:** 1 College of Food Sciences and Technology, Shanghai Ocean University, Shanghai 200120, China; 18332029749@163.com (Y.W.); 17356637955@163.com (L.H.); ly54329@163.com (Y.L.); 2 Shandong Marine Resource and Environment Research Institute, Yantai 264006, China; haotiantian0805@163.com (T.H.); szd892@126.com (Z.S.); wgmsd105@163.com (G.W.); 3 Yantai Key Laboratory of Quality and Safety Control and Deep Processing of Marine Food, Yantai 264006, China

**Keywords:** sea cucumber mouthpart peptides, gel beads, structural characterization, sustained-release performance, antioxidant activity

## Abstract

Sea cucumber mouthpart peptides (SCPs) are marine bioactive peptides with considerable application potential. However, their oral delivery remains challenging because of their poor stability and low intestinal bioavailability. To develop a delivery system for SCPs with gastric protection and pH-responsive intestinal sustained-release properties, double-layer gel beads (SCP-BMs) were fabricated through ionic gelation, with SCPs as the core material and sodium alginate (SA) and chitosan (CS) as the wall materials. The preparation conditions of the gel beads were optimized using single-factor experiments and response surface methodology (RSM). The optimized gel beads were then characterized for their morphology, thermal stability, *in vitro* gastrointestinal release behavior, and antioxidant activity. The results showed that the optimal preparation conditions were 1.67% (*w*/*v*) sodium alginate, 0.96% (*w*/*v*) chitosan, and 2.16% (*w*/*v*) CaCl_2_. Under the optimized conditions, the encapsulation efficiency (EE) reached 94.33%, significantly higher than that of the single-layer gel beads (SCP-SMs, 58.61%). Structural characterization showed that SCP-BMs exhibited a more compact structure than SCP-SMs, along with improved thermal stability. *In vitro* release and antioxidant assays demonstrated that SCP-BMs exhibited better gastric protection, pH-responsive intestinal release, and higher 2,2-diphenyl-1-picrylhydrazyl (DPPH) and 2,2′-azino-bis-3-ethylbenzothiazoline-6-sulphonic acid (ABTS) radical scavenging activity compared with SCP-SMs. This study demonstrated that the SA/CS double-layer wall material system effectively improved the encapsulation efficiency, structural stability, and intestinal release behavior of SCP gel beads. These findings provide a feasible strategy for the development of SCP delivery systems and offer a theoretical basis for the high-value utilization of sea cucumber byproducts.

## 1. Introduction

Sea cucumbers are valuable marine food resources due to their high nutritional value and diverse bioactive compounds. With the rapid expansion of sea cucumber fisheries and aquaculture worldwide, large quantities of byproducts, including viscera and mouthparts, are generated during processing. These byproducts are often discarded or underutilized, leading to substantial waste of resources and environmental burdens [1]. Current studies on the high-value utilization of sea cucumber byproducts have mainly focused on body wall collagen [2] and viscera polysaccharides [3], whereas the sea cucumber mouthpart remains largely underutilized. Compared with sea cucumber body wall and visceral tissues, the oral organ is a protein-rich digestive tissue and serves as a promising raw material for the preparation of bioactive peptides [4]. Studies have shown that sea cucumber peptides possess various physiological functions, including antioxidant, antihypertensive, hypoglycemic, antitumor, anti-inflammatory, and anti-fatigue activities [5,6,7,8,9,10]. However, research on the development and utilization of sea cucumber oral-derived peptides remains limited, and their structural characteristics, stability, and delivery systems have not been systematically investigated. Like most bioactive peptides, sea cucumber peptides exhibit limited stability and are susceptible to degradation during processing and storage due to environmental factors such as temperature and pH [11]. In addition, orally administered sea cucumber peptides are readily degraded in the gastrointestinal tract by gastric acid and pepsin, resulting in low bioavailability and restricted practical application [12]. Therefore, the development of a delivery system capable of protecting sea cucumber mouthpart peptides (SCPs) and achieving intestinal sustained release is of great significance for expanding their applications in the functional food field.

Encapsulation of bioactive peptides is an effective strategy for improving their stability and achieving controlled release. The protective matrix formed by wall materials can reduce environmental degradation and regulate the release behavior of encapsulated compounds, thereby improving their stability and potential biological efficacy [13]. Various encapsulation strategies have been developed for bioactive compounds, including ionic gelation [14], spray drying [15], and complex coacervation [16], among others. Among these approaches, ionic gelation is particularly attractive for preparing polymer-based gel beads. This technique relies on electrostatic interactions between charged polymers and crosslinking ions to form a three-dimensional gel network, enabling the formation of gel beads under mild processing conditions. Owing to its mild processing conditions, simple preparation, and excellent biocompatibility, this technique is widely used for encapsulating bioactive compounds, including peptides and probiotics [17]. Common polymer materials used for ionic gelation-based encapsulation include alginate, chitosan, gelatin, and pectin, among others [18]. Sodium alginate (SA) is one of the most widely used natural polysaccharides because of its excellent biocompatibility and ability to rapidly crosslink with Ca^2+^, forming a three-dimensional gel network. However, alginate gels alone generally exhibit a loose structure, limited mechanical strength, and poor acid resistance. As a result, they are susceptible to swelling and disintegration in gastric conditions, which limits their effectiveness for intestinal-targeted delivery [19]. Chitosan (CS) is a natural cationic polysaccharide. Its protonated amino groups can interact electrostatically with the carboxyl groups of sodium alginate to form a polyelectrolyte complex (PEC), thereby enhancing the density and acid resistance of the gel network [20]. Compared with single-layer wall materials, composite wall systems provide better control over the release behavior of encapsulated compounds and improve their protection and delivery efficiency. Previous studies have demonstrated the effectiveness of SA/CS composite systems for the delivery of bioactive compounds. For example, Lu et al. [21] successfully prepared SA/CS composite materials encapsulating tilapia collagen peptides, which achieved sustained release in the gastrointestinal tract, enhanced cell viability, and improved alcohol metabolism. Thuekeaw et al. [22] found that porous double-layer polymers made of SA and CS containing basil oil (BO) retained the antioxidant and antimicrobial activities of BO and exhibited tolerance to acid and bile. To the best of our knowledge, there are no reports on the preparation of SA/CS double-layer gel beads loaded with sea cucumber mouthpart peptides or their digestion and release behaviors.

In this study, SCPs were encapsulated into SA/CS double-layer gel beads via ionic gelation, with single-layer sodium alginate gel beads used as a control. The formulation was optimized using single-factor experiments and response surface methodology (RSM), followed by structural and thermal stability characterization. *In vitro* gastrointestinal digestion was performed to evaluate their release behavior and antioxidant activity. This study aimed to construct an intestinal sustained-release delivery system for sea cucumber mouthpart peptides and to provide a theoretical basis for the design of marine-derived peptide delivery systems as well as the high-value utilization of sea cucumber byproducts.

## 2. Results and Discussion

### 2.1. Optimization of the Preparation Process for SCP Gel Beads

#### 2.1.1. Single-Factor Experiments Analysis

**Figure 1:**
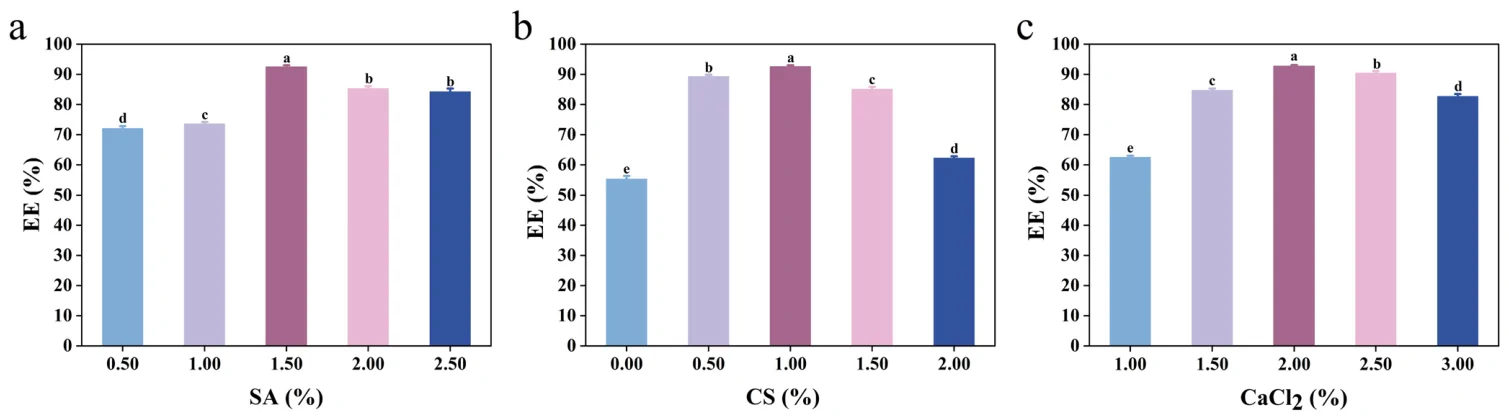
Single-factor experiments. (**a**) Effect of SA concentration on EE of SCP; (**b**) effect of CS concentration on EE of SCP; and (**c**) effect of CaCl_2_ concentration on EE of SCP. Different letters at top of columns indicate statistically significant differences (*p* < 0.05).

The single-factor effects of SA, CS, and CaCl_2_ concentrations on the encapsulation efficiency (EE) of SCP gel beads are presented in Figure 1.

EE exhibited an initial increase followed by a decrease as the SA concentration increased (Figure 1a). The highest EE was achieved at an SA concentration of 1.50% (*p* < 0.05). When the SA concentration was insufficient, the gel network formed after Ca^2+^ crosslinking was relatively loose, allowing SCPs to diffuse out of the matrix more readily. In addition, the weak network structure was prone to collapse during drying, resulting in a lower EE. Conversely, excessive SA concentrations increased the viscosity of the solution, resulting in larger droplets and pronounced tailing during gel bead formation. This led to non-uniform gel bead structures, as well as aggregation and breakage, ultimately reducing the EE [23]. Thus, under single-factor experimental conditions, a SA concentration of 1.50% was considered optimal.

As shown in Figure 1b, the EE of SCP gel beads increased significantly as the CS concentration increased from 0 to 1.00%. This can be attributed to the electrostatic interactions between the protonated amino groups on chitosan molecular chains and the dissociated carboxyl groups of sodium alginate, resulting in the formation of a polyelectrolyte complex (PEC). Together with Ca^2+^-mediated crosslinking, a dense composite capsule wall was established, effectively reducing the porosity of the gel network, minimizing core material leakage, and thereby enhancing EE. When the CS concentration exceeded 1.00%, the viscosity of the system increased, and excessive CS tended to induce local aggregation within the capsule wall, resulting in structural heterogeneity. Consequently, the number of gel bead formation defects increased, the stability of core material entrapment decreased, and the EE gradually declined. This trend is consistent with the findings of Atma et al. [24], who demonstrated that the incorporation of an appropriate amount of CS led to the formation of a polyelectrolyte complex, which refined the pore structure of the calcium alginate crosslinked network and reduced the porosity of the wall matrix. In summary, under the single-factor experimental conditions, the optimal concentration of CS was determined to be 1.00%.

EE exhibited an initial increase followed by a decrease as the CaCl_2_ concentration increased (Figure 1c). The maximum EE was obtained at a CaCl_2_ concentration of 2.00% (*p* < 0.05), indicating that an appropriate level of Ca^2+^ was essential for efficient gel bead formation. A low Ca^2+^ concentration resulted in insufficient crosslinking, producing a loose and porous network structure. This facilitated the leakage of SCPs during gelation and washing and weakened the interaction between SA and CS, thereby reducing the EE [25]. In contrast, excessively high Ca^2+^ concentrations can accelerate the crosslinking process, leading to excessive solidification of the outer gel layer and the formation of a dense shell, which hinders the effective entrapment of the core material. Furthermore, the increased ionic strength interfered with the formation of the SA-CS polyelectrolyte complex, resulting in non-uniform and fragile gel bead structures and consequently a lower EE [26]. Therefore, the optimal CaCl_2_ concentration was determined to be 2.00%.

#### 2.1.2. RSM Optimization Experiments

The factors, levels, and experimental results of the RSM optimization for SCP gel bead preparation are shown in Table 1 and Table 2. The experimental data were analyzed using Design-Expert 13 software, and the following quadratic regression equation was obtained:

(1) $$\begin{array}{c} Y\;=\;-10.868\;+\;16.8955A\;+\;7.367B\;+\;81.2865C\;+\;3.3AB-0.55AC\;+\;2.32BC-5.921A^{2}\;-9.131B^{2}-19.221C^{2} \end{array}$$

Analysis of variance (ANOVA) was performed to evaluate the adequacy of the regression model (Table 3). The model was highly significant (*p* < 0.0001), while the lack-of-fit was not significant (*p* = 0.0769), indicating that the model adequately described the experimental data. The R^2^ and R^2^adj values were 0.9975 and 0.9943, respectively, indicating an excellent agreement between the predicted and experimental values and demonstrating good reproducibility of the model. These results indicate that the regression model can accurately describe the effects of the three variables on EE and can be used to predict the optimal preparation conditions.

As shown in Table 3, the linear terms A (SA), B (CS), and C (CaCl_2_), as well as the interaction terms AB and BC, were significant factors influencing EE (*p* < 0.05). Based on the F-values, the influence of the three variables on the encapsulation efficiency of SCP gel beads decreased in the following order: C (CaCl_2_) > A (SA) > B (CS). These results suggest that CaCl_2_ concentration played the dominant role in determining the encapsulation efficiency of the gel beads.

**Figure 2:**
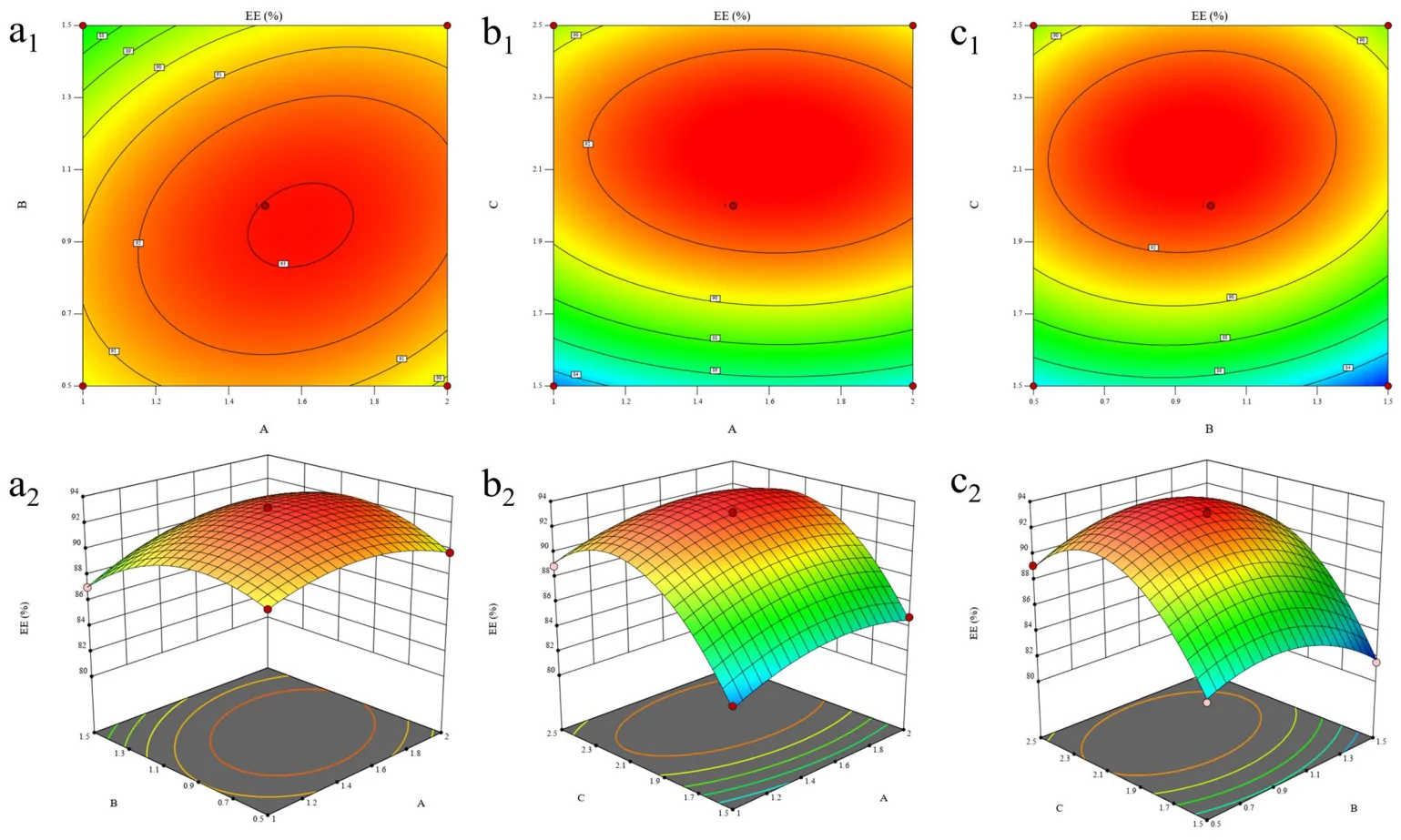
B curves and contour maps of factor interactions. (**a_1_**,**a_2_**) The effect of the interaction between SA (A) and CS (B); (**b_1_**,**b_2_**) the effect of the interaction between SA (A) and CaCl_2_ (C); and (**c_1_**,**c_2_**) the effect of the interaction between CS (B) and CaCl_2_ (C).

The three-dimensional (3D) response surface plots were used to visualize the interactions among the variables (Figure 2). Significant interaction effects were detected for AB and BC. The response surface of AB was steeper than those of the other interaction terms, indicating that the interaction between SA and CS had the greatest influence on EE. This observation agreed well with the ANOVA results, which identified AB as a highly significant interaction term (*p* < 0.01). In contrast, the interaction between SA and CaCl_2_ (AC) was not significant (*p* > 0.05). This may be attributed to the relatively stable formation of the gel network between SA and Ca^2+^. Within an appropriate range of CaCl_2_ concentrations, the available Ca^2+^ is sufficient to satisfy the crosslinking requirements between alginate molecular chains. Therefore, further variations in either SA or CaCl_2_ concentration do not substantially affect the ability of the gel network to entrap SCP [27].

The optimal formulation for SCP gel bead preparation was predicted using Design-Expert 13 software. The optimized conditions were an SA concentration of 1.67% (*w*/*v*), a CS concentration of 0.96% (*w*/*v*), and a CaCl_2_ concentration of 2.16% (*w*/*v*). Under these conditions, the predicted EE was 93.51%. Validation experiments conducted under the optimized conditions yielded an EE of 94.33%, which was in close agreement with the predicted value, confirming the accuracy and reliability of the model. In addition, blank sodium alginate gel beads (SM) and peptide-loaded sodium alginate gel beads (SCP-SM) were prepared under the same conditions. The EE of SCP-SMs was 58.61%, which was markedly lower than that of the SA/CS composite gel beads. This result was consistent with the single-factor experiments and demonstrated the superior encapsulation performance of the composite wall material system.

#### 2.1.3. Particle Size, EE, and Peptide Loading Capacity (LC) of Gel Beads

The average particle size, EE, and LC of the gel beads are summarized in Table 4. The average particle size of SCP-BMs (1.95 ± 0.11 mm) was larger than that of SCP-SMs (1.41 ± 0.14 mm), which may be attributed to the formation of an outer coating layer through electrostatic interactions between CS and SA, resulting in an increase in gel bead size [28]. The EE of SCP-BMs (94.33 ± 0.46%) was significantly higher than that of SCP-SMs (58.61 ± 0.92%), indicating that the SA/CS bilayer structure was more effective in reducing SCP loss during gel bead formation and consequently enhanced the retention of SCP within the gel beads. In addition, the LC of SCP-BMs (31.91 ± 0.79%) was higher than that of SCP-SMs (27.04 ± 1.23%). Although the incorporation of CS increased the amount of wall material, the substantial improvement in EE promoted greater retention of SCPs, thereby resulting in a higher LC. These results suggest that the SA/CS bilayer structure enhanced the EE of the gel beads while maintaining a high LC.

### 2.2. Structural Characterization of Gel Beads

#### 2.2.1. Microstructural Observation

**Figure 3:**
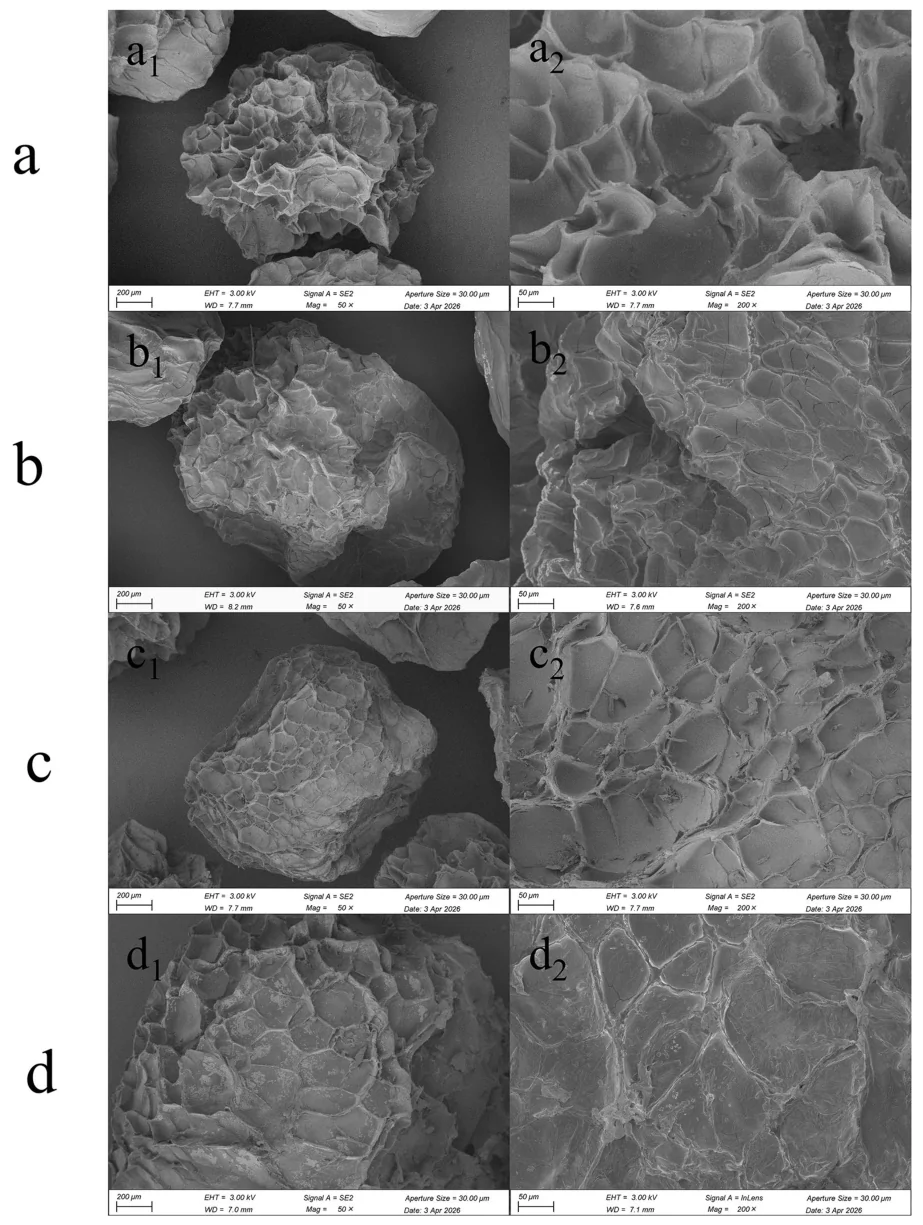
SEM of four types of gel beads. (**a**) SM; (**b**) SCP-SM; (**c**) BM; (**d**) SCP-BM (×50; ×200). (**a_1_**) SEM image of SM at ×50 magnification; (**a_2_**) SEM image of SM at ×200 magnification; (**b_1_**) SEM image of SCP-SM at ×50 magnification; (**b_2_**) SEM image of SCP-SM at ×200 magnification; (**c_1_**) SEM image of BM at ×50 magnification; (**c_2_**) SEM image of BM at ×200 magnification; (**d_1_**) SEM image of SCP-BM at ×50 magnification; and (**d_2_**) SEM image of SCP-BM at ×200 magnification.

The surface morphology of the different gel beads was characterized by scanning electron microscopy (SEM) (Figure 3). In general, all four types of gel beads exhibited irregular spherical morphologies with wrinkled surfaces, which may be attributed to water removal and structural shrinkage during freeze-drying. The single-layer gel beads (SM and SCP-SM) exhibited pronounced honeycomb-like pores and surface depressions, whereas the double-layer gel beads (BM and SCP-BM) displayed denser surface structures with substantially fewer pores. Compared with the blank gel beads, the peptide-loaded gel beads exhibited finer surface wrinkles. Among them, SCP-BM showed the smoothest and most intact surface morphology. This observation suggests that the incorporation of CS promoted the formation of a denser outer layer, resulting in a smoother capsule surface. In addition, the electrostatic interaction between CS and SA may have enhanced the hydrophobicity and structural stability of the wall materials [29]. The synergistic effect of these interactions led to the formation of a more compact and homogeneous gel bead structure, which contributed to the improved EE and stability of SCPs.

#### 2.2.2. Fourier Transform Infrared (FTIR) Analysis

**Figure 4:**
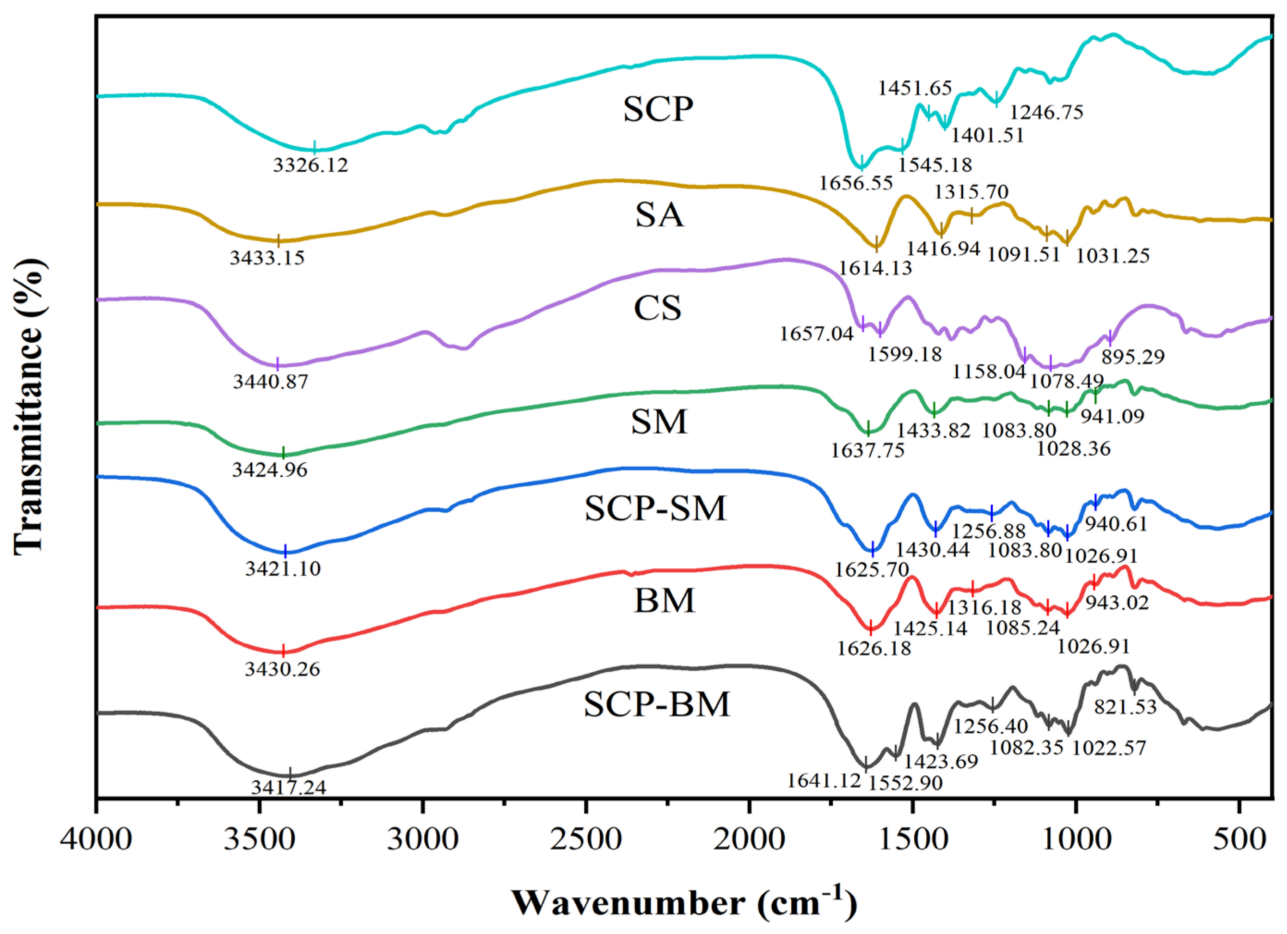
FTIR spectra of SCP, SA, CS, SM, SCP-SM, BM, and SCP-BM.

FTIR spectroscopy was employed to verify the successful encapsulation of SCPs and to elucidate the intermolecular interactions among the gel bead components. The FTIR spectra of SCPs, SA, CS, SMs, SCP-SMs, BMs, and SCP-BMs are presented in Figure 4. According to previous reports, the absorption band around 3400 cm^−1^ is attributed to the stretching vibrations of hydroxyl (-OH) and amino (-NH) groups [30]. The absorption band in the range of 1600–1700 cm^−1^ is assigned to the amide I band and is mainly attributed to the stretching vibration of the carbonyl group (C=O) in peptide bonds (-CO-NH-). The characteristic band in the range of 1500–1600 cm^−1^ corresponds to the amide II band, arising mainly from N-H bending and C-N stretching vibrations [31]. As shown in Figure 4, the broad absorption band around 3400 cm^−1^, corresponding to the overlapping stretching vibrations of hydroxyl (O–H) and amino (N–H) groups, exhibited a slight shift after SCP encapsulation. This change suggests enhanced intermolecular interactions, including hydrogen bonding among SCPs, SA, and CS, which, together with electrostatic interactions, contributed to the formation of a more stable polyelectrolyte network within the bilayer gel beads [32]. In addition, SCPs exhibited characteristic absorption peaks at 1656 and 1545 cm^−1^, which were assigned to the C=O stretching vibration of peptide bonds (amide I) and the amide II band, respectively. The absorption bands observed at 1637 cm^−1^ in SM and 1626 cm^−1^ in BMs are primarily attributed to the asymmetric stretching vibrations of carboxylate groups (COO^−^) in sodium alginate, although slight shifts may occur due to Ca^2+^ crosslinking and interactions within the gel network [33]. Following SCP incorporation, the corresponding band in SCP-BMs shifted to 1641 cm^−1^. This shift may be associated with the formation and structural rearrangement of the SA/CS polyelectrolyte complex, in which electrostatic interactions between the carboxylate groups of SA (1610–1630 cm^−1^) and the protonated amino groups of CS contribute to the observed spectral features. In addition, the incorporation of SCPs within the alginate matrix may further modulate the local hydrogen-bonding and electrostatic microenvironment, thereby contributing to the observed spectral changes [34]. These findings support the successful incorporation of SCPs into the SA/CS double-layer gel beads. Furthermore, the observed spectral changes suggest the presence of intermolecular interactions within the double-layer matrix, which may contribute to the protective properties of the encapsulation system.

#### 2.2.3. X-Ray Diffraction Analysis (XRD)

**Figure 5:**
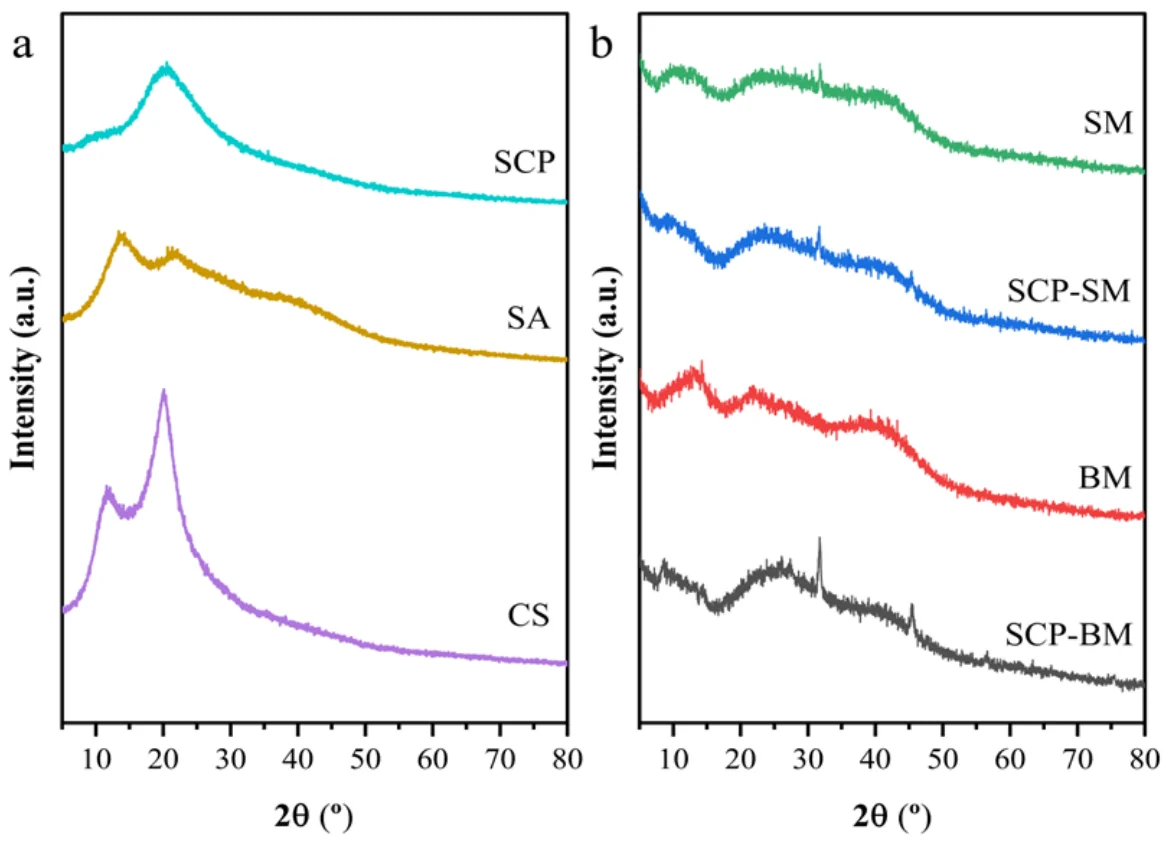
XRD patterns. (**a**) XRD patterns of SCP, SA, and CS; (**b**) XRD patterns of SM, SCP-SM, BM, and SCP-BM.

XRD analysis was employed to investigate the crystalline structures of the raw materials and gel beads. The XRD patterns of SCPs, SA, and CS are shown in Figure 5a, whereas those of SMs, SCP-SMs, BMs, and SCP-BMs are presented in Figure 5b. SCPs exhibited a broad diffraction peak at approximately 2θ = 20.9°, suggesting that it predominantly possessed an amorphous structure [35]. SA exhibited characteristic diffraction peaks at 2θ = 13.9° and 21.3°, whereas CS displayed characteristic peaks at 2θ = 11.9° and 20.0°, indicating the presence of ordered crystalline regions in their structures [36]. Compared with SA, the diffraction peak intensities of SMs and SCP-SMs at 2θ = 13.9° and 21.3° were markedly reduced, indicating a decrease in structural ordering. This reduction may be partially attributed to intermolecular interactions within the gel bead matrix, which could disrupt the ordered arrangement of SA chains [37]. Similarly, the characteristic diffraction peaks of CS near 2θ = 11.9° and 20.0° became markedly less pronounced in BMs and SCP-BMs. Since BMs and SCP-BMs are bilayer beads with an alginate core and chitosan shell, the marked decrease in chitosan-related peak intensities can be largely attributed to physical dilution of chitosan by the alginate core, with additional contribution from interfacial electrostatic interactions that partially disrupt chitosan chain ordering. Therefore, the reduced crystallinity is likely a synergistic outcome of both dilution and chemical interactions, rather than solely due to electrostatic forces [38]. In addition, new diffraction peaks appeared near 2θ = 31.8° and 40.5° in SMs, SCP-SMs, BMs, and SCP-BMs, whereas these peaks were absent in the raw wall materials. These reflections are more likely associated with ionically induced ordering within the Ca^2+^-crosslinked alginate network and possible formation of localized ordered domains during the ion gelation process [39]. No characteristic crystalline peaks attributable to SCP were observed in either SCP-SMs or SCP-BMs. This result indicates that SCPs were successfully encapsulated within the gel beads and predominantly existed in an amorphous or molecularly dispersed form. A similar phenomenon was reported by Li et al. [40], who found that the characteristic diffraction peaks of squid pen trypsin hydrolysates disappeared after microencapsulation, suggesting that the peptides were successfully incorporated into the gel bead matrix and predominantly existed in an amorphous state. The consistency between their findings and the present results further supported our interpretation of the XRD data. Overall, the XRD results suggest that SCPs were incorporated into the gel bead matrix and that the ion gelation process altered the structural ordering of the polymer network.

#### 2.2.4. Thermal Stability Analysis

**Figure 6:**
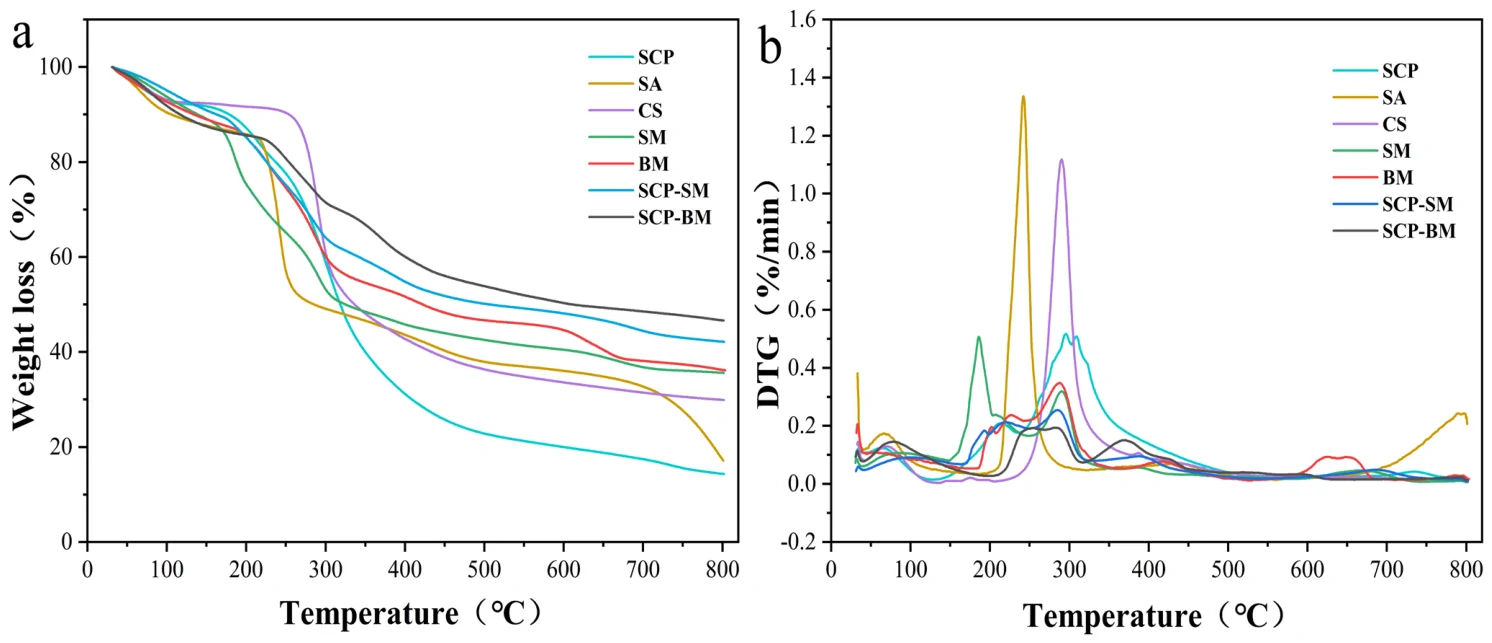
TG and DTG curves (**a**) TG curves; (**b**) DTG curves.

The thermal stability of the gel beads was evaluated using thermogravimetric (TG) (Figure 6a) and derivative thermogravimetric (DTG) analyses (Figure 6b). As shown in Figure 6, SCPs exhibited a pronounced weight loss peak at 309.2 °C, with a maximum weight loss rate of 0.51%/min. This peak was primarily associated with peptide bond cleavage and the thermal degradation of the protein backbone. During the main degradation stage (158–350 °C), SCPs exhibited a mass loss of 51.57%, indicating limited thermal stability [41]. Following encapsulation in the single-layer gel beads (SCP-SMs), the maximum weight loss rate decreased from 0.51 to 0.26%/min, while the mass loss during the main degradation stage (158–350 °C) decreased to 30.80%. This value was considerably lower than those of SCPs (51.57%) and SMs (39.66%). These findings suggest that interactions between SCPs and the SA matrix restricted molecular-chain mobility and increased the energy required for thermal degradation, thereby improving thermal stability [42]. A more pronounced improvement in thermal stability was observed for SCP-BMs. Although the main thermal degradation peak was detected at 284.4 °C, SCP-BMs exhibited the lowest maximum weight loss rate (0.19%/min) among all peptide-loaded gel beads. In addition, SCP-BMs exhibited a mass loss of only 20.26% during the main thermal degradation stage (158–350 °C), which was substantially lower than that of SCP-SMs (30.80%). This result suggests that the multiple intermolecular interactions and the compact bilayer structure more effectively retarded the thermal degradation of peptide chains [43]. In the high-temperature range of 500–800 °C, SCP-BMs exhibited a lower mass loss (7.10%) than SCP-SMs (7.82%). This behavior may be attributed to the nitrogen-containing functional groups in CS, which facilitated the formation of thermally stable nitrogen-doped carbonaceous structures and inhibited the further oxidative decomposition of the residual char [44].

In addition, SCP-BMs exhibited a higher mass loss (13.07%) from room temperature to 158 °C than SCP-SMs (9.91%) and the individual components. This result may be attributed to the abundant hydrophilic groups and enhanced water-binding capacity of the bilayer SA-CS network, which promoted the retention of physically adsorbed and bound water within the gel bead matrix [45]. In the temperature range of 350–500 °C, SCP-BMs exhibited a slightly higher mass loss (12.85%) than SCP-SMs (9.30%). This behavior may be associated with the decomposition of nitrogen-containing functional groups in CS and the subsequent release of volatile nitrogenous species, including NH_3_ and HCN [44]. Collectively, the thermogravimetric results demonstrated that SCP-BMs possessed superior thermal stability and thermal protection capability compared with SCP-SMs. These findings suggest that the bilayer gel bead system is a more promising carrier for the delivery and protection of SCPs.

### 2.3. In Vitro Release Analysis

**Figure 7:**
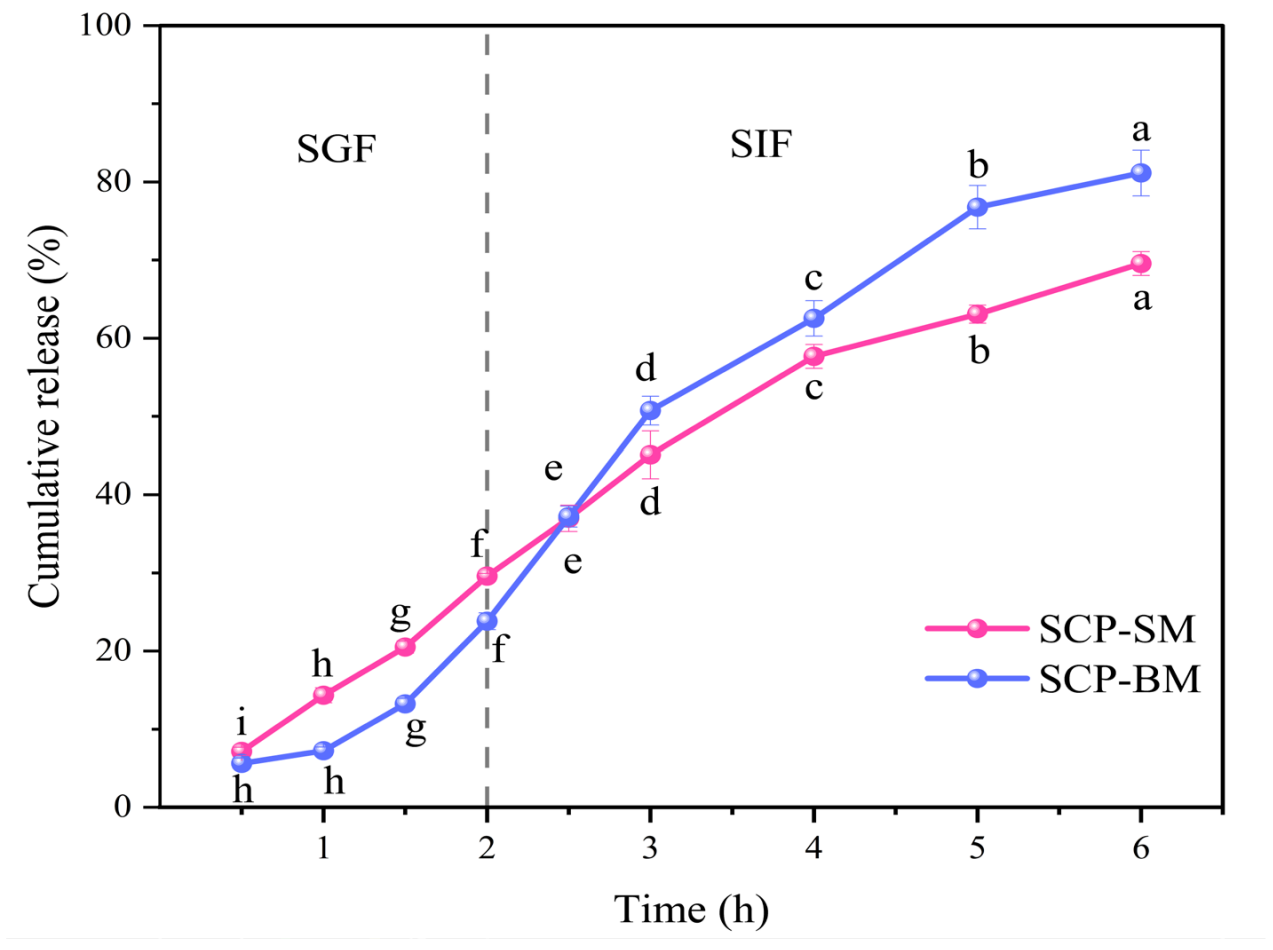
Cumulative release profiles of SCP-SM and SCP-BM during simulated gastrointestinal digestion. Different letters indicate significant differences (*p* < 0.05).

*In vitro* simulated gastrointestinal digestion experiments were conducted to evaluate the protective effect of the gel beads on SCPs and their intestinal release behavior, providing experimental evidence for improving the gastrointestinal delivery performance of SCPs. The cumulative release profiles of SCPs from SCP-SMs and SCP-BMs during simulated gastrointestinal digestion are shown in Figure 7. During the simulated gastric fluid (SGF, 0–2 h) stage, both gel beads exhibited a slow-release behavior, with SCP-SMs showing a slightly higher release rate than SCP-BMs. After 2 h of digestion, the cumulative release rates reached 29.59% and 23.83%, respectively. This phenomenon may be attributed to the dense network formed by polyelectrolyte complexation between sodium alginate and chitosan in the bilayer structure of SCP-BMs. Under acidic conditions, protonation of the amino groups of chitosan (-NH_3_^+^) strengthened their electrostatic interactions with the carboxyl groups (-COO^−^) of sodium alginate, thereby enhancing the mechanical strength of the gel beads and suppressing the release of sea cucumber peptides [46]. In contrast, SCP-SMs relied solely on the ionically crosslinked sodium alginate network. Partial protonation of the carboxyl groups under gastric conditions resulted in a looser gel network, facilitating the diffusion and release of peptide molecules. Wang et al. [47] also observed a similar phenomenon, indicating that bilayer gel beads exhibited superior structural stability and acid resistance under acidic conditions compared with single-layer gel beads.

Upon transition to the simulated intestinal fluid (SIF, 2–6 h) stage, the release rate of SCPs increased markedly, and the release rate of SCP-BMs was consistently higher than that of SCP-SMs at all sampling time points. At the end of the 6 h digestion period, the cumulative release rate of SCP-BM reached 81.14%, which was significantly higher than that of SCP-SM (69.56%) (*p* < 0.05). This phenomenon was associated with the pH of the release medium. Under neutral intestinal conditions, deprotonation of chitosan weakened the electrostatic interactions within the SA/CS polyelectrolyte complex, leading to partial structural relaxation of the bilayer network. Meanwhile, the carboxyl groups of SA dissociated into -COO^−^ under neutral conditions, leading to swelling of the gel network and facilitating peptide diffusion [48]. For SCP-BM, the relaxation of the outer CS network and the swelling of the inner SA layer acted synergistically in the intestinal environment, creating more accessible diffusion pathways and accelerating peptide release. In contrast, the monolayer SCP-SM structure had already undergone partial swelling during the gastric phase. Although further swelling occurred upon exposure to intestinal fluid, the absence of a pH-responsive dissociation mechanism associated with CS limited the increase in its release rate, and part of the peptide cargo may have been lost due to premature release during the gastric stage. This result suggests that SCP-BMs effectively delayed SCP release in the gastric phase while enabling enhanced release in the intestinal phase, exhibiting a pronounced pH-responsive release behavior. Compared with the single-layer system, the SA/CS bilayer provided enhanced gastric protection while promoting pH-triggered intestinal release, providing a theoretical basis for the development of an intestinal delivery system for SCPs.

### 2.4. Antioxidant Activity

**Figure 8:**
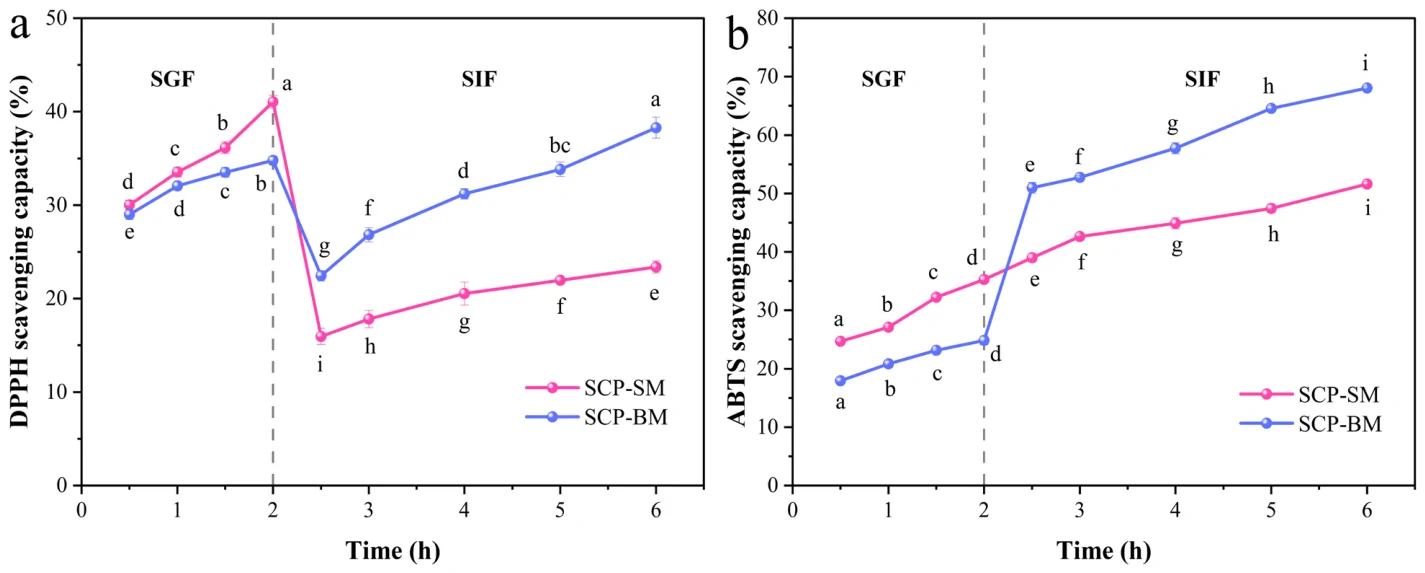
DPPH and ABTS radical scavenging rate of SCP-SM and SCP-BM after simulated gastrointestinal digestion. (**a**) DPPH radical scavenging rate of SCP-SM and SCP-BM after simulated gastrointestinal digestion; (**b**) ABTS radical scavenging rate of SCP-SM and SCP-BM after simulated gastrointestinal digestion. Different letters indicate significant differences among different time points within same group (*p* < 0.05).

As shown in Figure 8, both SCP-SMs and SCP-BMs exhibited antioxidant activity after digestion. However, their free radical scavenging capacities varied with digestion time. Moreover, SCP-BMs showed more stable bioactive release behavior across different digestion stages.

During the simulated gastric digestion stage, both SCP-SMs and SCP-BMs exhibited relatively weak DPPH and ABTS radical scavenging activities. However, as digestion progressed, SCP was gradually released, which may contribute to the increased scavenging capacities against both radicals. Within the first 2 h of simulated gastric digestion, the DPPH and ABTS radical scavenging activities of SCP-SMs increased rapidly and reached maximum values of 41.06% and 35.30%, respectively. In contrast, the antioxidant activities of SCP-BM increased more gradually, reaching maximum values of 34.78% and 24.85%, respectively. The lower antioxidant activity of SCP-BMs during the gastric phase may be attributed to its more compact gel bead structure; however, this dense barrier could effectively protect SCP from excessive degradation by pepsin and the highly acidic gastric environment [49]. During the simulated intestinal digestion stage (2–6 h), the DPPH radical scavenging activities of both SCP-SMs and SCP-BMs were generally lower than those observed during the gastric phase, although a gradual increasing trend was still evident, reaching 23.39% and 38.29%, respectively. The decreased DPPH scavenging activity despite increased peptide release suggests that peptide availability alone may not determine antioxidant capacity. This phenomenon may be related to further enzymatic hydrolysis of released SCPs, which alters peptide sequences and molecular weight distribution, potentially degrading some antioxidant-active peptides while generating new peptides with different activities. Therefore, increased peptide availability does not necessarily lead to a proportional enhancement of DPPH scavenging activity [50]. In contrast, the ABTS radical scavenging activities of both gel beads during the simulated intestinal digestion stage were generally higher than those during the gastric phase. The ABTS radical scavenging activities of SCP-SMs and SCP-BMs increased significantly with digestion time. Notably, the ABTS radical scavenging activity of SCP-BMs increased sharply after transfer to simulated intestinal fluid. After 6 h of simulated intestinal digestion, the ABTS radical scavenging activities of SCP-SMs and SCP-BMs reached 51.63% and 68.06%, respectively. The different trends observed between DPPH and ABTS assays may be attributed to their distinct reaction mechanisms and sensitivities toward antioxidant structures. Therefore, peptide transformation during intestinal digestion may affect DPPH and ABTS scavenging capacities differently [51]. These results suggest that, upon entering the intestinal environment, the increase in pH promoted swelling of the SCP-BM gel beads, facilitating the gradual release of SCPs and contributing to the maintenance of antioxidant activity. However, peptide transformation during gastrointestinal digestion, including the generation of smaller peptide fragments with potential antioxidant properties, may also contribute to the observed radical scavenging activity. This result is consistent with the findings of Xu et al. [52], who reported that microencapsulation can reduce environmental interference during gastrointestinal digestion through the protective effect of wall materials, thereby enabling more stable peptide release behavior. Compared with SCP-SMs, SCP-BMs showed stronger pH-responsive peptide delivery during simulated gastrointestinal digestion. It also better maintained its antioxidant activity. These results indicate its potential for application in marine-derived bioactive peptide delivery systems.

## 3. Materials and Methods

### 3.1. Materials

The dried sea cucumber mouthpart (SCP) was purchased from Yantai Wanshiruyi Food Co., Ltd. (Yantai, China). Papain (enzyme activity 1000 U/mg) was purchased from Nanning Pangbo Bioengineering Co., Ltd. (Nanning, China). Pepsin (porcine origin, enzyme activity 3000 U/mg) and trypsin (porcine origin, enzyme activity 250 U/mg) were purchased from Beijing Solarbio Biotechnology Co., Ltd. (Beijing, China). 1,1-Diphenyl-2-picrylhydrazyl (DPPH) and 2,2′-azinobis-(3-ethylbenzothiazoline-6-sulfonate) ammonium salt (ABTS) were obtained from Shanghai Yuanye Bio-Technology Co., Ltd. (Shanghai, China). Sodium alginate, calcium chloride, chitosan (degree of deacetylation > 99%), potassium persulfate, and other reagents were purchased from Sinopharm Chemical Reagent Co., Ltd. (Shanghai, China) and were of analytical grade.

### 3.2. Preparation of SCP

Dried SCP (10.00 g) was dispersed in 90 mL of deionized water at a solid-to-liquid ratio of 1:9 (*w*/*v*) and rehydrated overnight at 4 °C. After removing impurities, the sample was homogenized and diluted with deionized water at a volume ratio of 1:20 (*v*/*v*). Papain (2%, *w*/*w*) was then added, and the mixture was incubated in a water bath at 55 °C for 6 h to allow for enzymatic hydrolysis. After the reaction, the enzymatic activity was terminated by heating in a water bath at 100 °C for 10 min to effectively inactivate papain and prevent excessive hydrolysis. The mixture was then centrifuged at 4 °C and 5000 rpm for 15 min, and the supernatant was collected and sequentially filtered through 1.0, 0.45, and 0.22 µm membrane filters. The filtrate was subjected to tangential flow ultrafiltration using a 3 kDa molecular weight cut-off (MWCO) membrane, and the permeate fraction (<3 kDa) was collected. The collected permeate was subsequently dialyzed using 1 kDa dialysis tubing for 24 h for desalting and purification. The resulting solution was further concentrated by rotary evaporation and subsequently lyophilized to obtain SCP powder. The lyophilized powder was stored at −20 °C in the dark prior to further ionic gelation-based encapsulation and activity evaluation. Molecular weight distribution analysis showed that 97.70% of the peptides had molecular weights below 1.5 kDa.

### 3.3. Preparation of Gel Beads

A predetermined amount of sodium alginate was dissolved in 50 mL of distilled water under magnetic stirring for 2 h to prepare the sodium alginate solution, which served as the inner wall material. Separately, a predetermined amount of chitosan was dissolved in 1% (*v*/*v*) acetic acid solution and stirred in a water bath at 50 °C until completely dissolved, serving as the outer wall material. CaCl_2_ solution at the desired concentration was used as the coagulation solution. Subsequently, 1.00 g of SCP lyophilized powder was accurately weighed and uniformly dispersed in the sodium alginate solution. Before the coating process, 50 mL of the CS was mixed with 50 mL of the CaCl_2_ solution under continuous stirring to freshly prepare the chitosan-containing coagulation bath. The prepared coagulation bath was immediately used for gel bead preparation to minimize potential chitosan instability caused by pH variation. The SCP-containing sodium alginate solution was then loaded into a syringe equipped with a 25G needle and slowly dropped into the coagulation bath at a flow rate of 1 mL/min. After solidification for 30 min at 100 rpm, the resulting gel beads were collected by filtration, washed, and freeze-dried to obtain sea cucumber mouthpart peptide-loaded double-layer gel beads (SCP-BMs). Blank double-layer gel beads (BMs) without the SCP were prepared by directly dripping sodium alginate solution into a mixed coagulation bath. A syringe was used to drop sodium alginate solution containing 1.00 g of SCP lyophilized powder into 100 mL of CaCl_2_ solution to prepare sea cucumber mouthpart peptide single-layer gel beads (SCP-SM). Blank single-layer gel beads (SMs) were prepared by directly dripping sodium alginate solution without the SCP into the coagulation solution.

### 3.4. Determination of Encapsulation Efficiency (EE) and Peptide Loading Capacity (LC)

The EE was determined using the Bradford assay according to a previously reported method [53] with slight modifications. Briefly, 400 μL of the filtrate obtained from the freshly prepared peptide-loaded wet gel beads was mixed thoroughly with 2 mL of Coomassie Brilliant Blue solution, and the absorbance at 595 nm was measured within 5 min for $A_{1}$. Similarly, the filtrate from blank wet gel beads was analyzed to obtain the absorbance value $A_{2}$. Subsequently, 1.00 g of peptide was thoroughly mixed with 100 mL of coagulation bath solution, and the absorbance of the resulting mixture was determined by the Coomassie Brilliant Blue assay to obtain $A_{3}$. The absorbance of the blank coagulation bath was recorded as $A_{4}$. Encapsulation efficiency was expressed as the relative peptide loading rate and calculated according to the following equation:

(2) $$\mathrm{EE}\;(\%)\;=\;(1-\frac{A_{1}-A_{2}}{A_{3}-A_{4}})\;\times \;100$$

where $A_{1}$—absorbance of the residual solution from the peptide-loaded gel beads; $A_{2}$—absorbance of the residual solution from the blank gel beads; $A_{3}$—absorbance of the mixture of 1.00 g SCP and the coagulation bath; $A_{4}$—absorbance of the blank coagulation bath.

(3) $$\mathrm{LC}\;(g/g)\;=\frac{\mathrm{total}\;\mathrm{amount}\;\mathrm{of}\;\mathrm{encapsulated}\;\mathrm{peptide}\;\times \;\mathrm{EE}}{\mathrm{total}\;\mathrm{gel}\;\mathrm{bead}\;\mathrm{weight}}$$

It should be noted that the Bradford assay was employed in this study as a practical and comparative method for evaluating peptide encapsulation efficiency and loading capacity. Given that the SCP predominantly consists of low-molecular-weight peptides (<1.5 kDa), some peptide species may exhibit limited binding affinity toward Coomassie Brilliant Blue, potentially affecting absolute quantification. Therefore, the values reported in this study should be interpreted primarily for comparative purposes among different formulations rather than as absolute measurements.

### 3.5. Optimization of Gel Bead Preparation Conditions

#### 3.5.1. Experimental Design for a Single Factor

Based on preliminary experiments, the concentrations (*w*/*v*) of sodium alginate, chitosan, and CaCl_2_ solution were selected as factors to be optimized. A single-factor experiment was designed to evaluate the effects of sodium alginate concentration (0.50%, 1.00%, 1.50%, 2.00%, 2.50%), chitosan concentration (0, 0.50%, 1.00%, 1.50%, 2.00%), and CaCl_2_ solution concentration (1.00%, 1.50%, 2.00%, 2.50%, 3.00%) on EE.

#### 3.5.2. Experimental Design for RSM

Based on the results of the single-factor experiments, encapsulation efficiency was used as the response variable, and the concentrations of sodium alginate, chitosan, and CaCl_2_ solution were selected as the three independent variables. A three-factor, three-level Box–Behnken design was then performed using Design-Expert 13 software. The factors and their levels are presented in Table 1.

### 3.6. Characterization of the Structure and Morphology of Gel Beads

#### 3.6.1. Morphological Characterization

The morphology of the gel beads was characterized by scanning electron microscopy (SEM) (ZEISS Sigma 360 scanning electron microscope, Carl Zeiss AG, Oberkochen, Baden-Württemberg, Germany). An appropriate amount of freeze-dried gel beads was mounted on conductive adhesive tape and sputter-coated with gold using a sputter coater (Quorum SC7620, Quorum Technologies Ltd., East Sussex, UK) at 10 mA for 45 s. The microstructure and particle size of the samples were observed and recorded under an accelerating voltage of 3 kV.

#### 3.6.2. FTIR Analysis

An appropriate amount of freeze-dried gel beads was mixed and ground with dried potassium bromide (KBr) powder at a mass ratio of approximately 1:100. The thoroughly ground mixture was then compressed into transparent pellets and analyzed by Fourier transform infrared (FTIR) spectroscopy (Thermo Scientific Nicolet iS20 spectrometer, Thermo Fisher Scientific Inc., Waltham, MA, USA). Spectra were recorded over a wavenumber range of 4000–400 cm^−1^ at a resolution of 4 cm^−1^.

#### 3.6.3. XRD Analysis

The crystalline structure of the freeze-dried gel beads was analyzed by X-ray diffraction (XRD) (Bruker D8 Advance X-ray diffractometer, Bruker Corporation, Karlsruhe, Baden-Württemberg, Germany). The powdered samples were evenly spread on a glass sample holder prior to analysis. Measurements were performed using Cu Kα radiation with a tube voltage of 40 kV and a tube current of 40 mA. The diffraction patterns were recorded over a 2θ range of 5–80° at a scanning rate of 2°/min.

#### 3.6.4. Thermogravimetric Analysis (TGA)

Thermogravimetric analysis (TGA) of the freeze-dried gel beads was performed using a thermogravimetric analyzer (HITACHI STA200, Hitachi High-Tech Corporation, Tokyo, Japan). The samples were heated from 30 to 800 °C at a rate of 10 °C/min under a nitrogen atmosphere.

### 3.7. In Vitro Gastrointestinal Digestion of Gel Beads

#### 3.7.1. *In Vitro* Simulated Gastrointestinal Digestion

The simulated gastrointestinal fluids were prepared according to the method described by Chen et al. [54]. Simulated gastric fluid (SGF) was prepared by dissolving 0.2 g of NaCl in distilled water, adjusting the pH to approximately 1.2 with HCl, adding 0.32 g of pepsin, and then making up the volume to 100 mL. Simulated intestinal fluid (SIF) was prepared by dissolving 0.68 g of potassium dihydrogen phosphate (KH_2_PO_4_) in distilled water, adjusting the pH to 7.5 with NaOH, adding 1.0 g of trypsin, and then making up the volume to 100 mL.

The *in vitro* simulated gastrointestinal digestion assay was performed according to the method of Yuan et al. [53] with slight modifications. Briefly, 0.5 g of peptide-loaded gel beads and the corresponding blank gel beads were weighed separately, and the equivalent amount of free peptides was calculated based on the peptide loading capacity. The samples were suspended in 50 mL of SGF and incubated at 37 °C under continuous stirring at 100 rpm for 2 h. Aliquots were collected every 0.5 h and centrifuged at 4000 rpm for 5 min. The supernatants were collected, and their absorbance values were determined as described in Section 3.4. After each sampling, the volume was restored to 50 mL with fresh SGF. Following 2 h of gastric digestion, the gel beads were collected by filtration, rinsed with distilled water to remove residual pepsin and peptides, and gently blotted to remove excess moisture. The samples were then transferred into 50 mL of SIF for further digestion. Aliquots were collected after 0.5, 1, 1.5, 2, 3, and 4 h of intestinal digestion. The subsequent procedures were identical to those used during the gastric digestion stage.

#### 3.7.2. Cumulative Release Profile of SCP During *In Vitro* Digestion

The relative release at each of the above time points was calculated separately.

(4) $$\mathrm{relative}\;\mathrm{release}\;(\%)\;=\frac{A_{1}-A_{2}}{A_{3}-{\;A}_{4}}\;\times \;100$$

where $A_{1}$—absorbance of the supernatant from peptide-loaded gel beads in simulated gastrointestinal fluids at the corresponding time point; $A_{2}$—absorbance of the supernatant from blank gel beads in simulated gastrointestinal fluids at the corresponding time point; $A_{3}$—absorbance of the supernatant from the equivalent amount of free peptides in simulated gastrointestinal fluids at the corresponding time point; $A_{4}$—absorbance of the supernatant from blank simulated gastrointestinal fluids at the corresponding time point.

The release profiles of the peptide-loaded gel beads were constructed by plotting cumulative relative release against digestion time. All measurements were performed in triplicate.

### 3.8. Determination of Antioxidant Activity After In Vitro Simulated Gastrointestinal Digestion

#### 3.8.1. DPPH Radical Scavenging Activity

The DPPH radical scavenging activity was measured according to the method of Wang et al. [55] with slight modifications. DPPH solution (0.2 mmol/L) was prepared in absolute ethanol. The gastrointestinal fluid samples collected during the sustained release of the gel beads were mixed with the DPPH solution at a 1:1 ratio and incubated at room temperature in the dark for 30 min. The absorbance was then measured at 517 nm and recorded as $A_{1}$. Control samples were prepared by mixing absolute ethanol with gastrointestinal fluids at a 1:1 ratio to obtain $A_{2}$ and absolute ethanol with DPPH solution at a 1:1 ratio to obtain $A_{3}$. Each measurement was performed in triplicate, and the mean values were used for further calculations. The calculation formula is as follows:

(5) $$\mathrm{DPPH}\;\mathrm{scavenging}\;\mathrm{rate}\;(\%)\;=\;(1-\frac{A_{1}-A_{2}}{A_{3}})\;\times \;100$$

#### 3.8.2. ABTS Radical Scavenging Activity

The ABTS radical scavenging activity was determined according to the method of Fu et al. [56] with slight modifications. Briefly, 7 mmol/L ABTS solution and 2.45 mmol/L potassium persulfate solution were prepared separately and mixed at an equal volume ratio. The mixture was allowed to stand in the dark at room temperature for 12–16 h to obtain the ABTS^+^ stock solution. The stock solution was then diluted with absolute ethanol until an absorbance of 0.70 ± 0.02 at 734 nm was achieved, yielding the ABTS^+^ working solution. Subsequently, 100 μL of gastrointestinal fluid was mixed with 4 mL of ABTS^+^ working solution and incubated in the dark for 6 min. The absorbance was measured at 734 nm and recorded as $A_{1}$. Meanwhile, 100 μL of gastrointestinal fluid was mixed with 4 mL of absolute ethanol, and the absorbance was recorded as $A_{2}$. In addition, 100 μL of absolute ethanol was mixed with 4 mL of ABTS^+^ working solution, and the absorbance was recorded as $A_{3}$. Each measurement was performed in triplicate, and the mean value was used for calculation. The calculation formula is as follows:

(6) $$\mathrm{ABTS}\;\mathrm{scavenging}\;\mathrm{rate}\;(\%)\;=\;(1-\frac{A_{1}-A_{2}}{A_{3}})\;\times \;100$$

### 3.9. Statistical Analyses

All data are presented as the mean ± standard deviation (SD) of three independent experiments. Graphs were generated using Origin 2026 (OriginLab Corporation, Northampton, MA, USA). The response surface optimization data were analyzed by quadratic regression and ANOVA using Design-Expert 13 software (Stat-Ease Inc., Minneapolis, MN, USA), and statistical significance was evaluated using IBM SPSS Statistics 27 (IBM Corp., Armonk, NY, USA) (*p* < 0.01 indicates extremely significant differences; *p* < 0.05 indicates significant differences; and *p* > 0.05 indicates no significant difference.)

## 4. Conclusions

This study used SCPs as the core material and SA and CS as wall materials. Double-layer gel beads (SCP-BMs) were successfully prepared using the ion gelation method. The optimal preparation conditions were determined through single-factor experiments and response surface methodology. The double-layer wall material system significantly improved the encapsulation efficiency. Structural characterization confirmed that SCPs were successfully encapsulated within SCP-BMs. SCP-BMs exhibited a more compact and intact structure than SCP-SMs. Thermal stability analysis further showed that the double-layer structure significantly enhanced thermal stability. *In vitro* digestion experiments indicated that SCP-BMs exhibited gastric protection and pH-triggered intestinal release and better maintained the antioxidant activity of SCPs. Overall, the SA/CS bilayer system showed potential as a gastric-protective and intestinal-targeted delivery system for SCPs. It also showed superior stability compared with the single-layer system. This work provides a theoretical basis for developing pH-responsive gastrointestinal delivery systems for marine bioactive peptides and the high-value utilization of sea cucumber byproducts. However, the system’s industrial application is still limited by particle size control, scale-up production, and process stability. Moreover, the potential influence of upstream processing conditions, such as enzyme termination strategies, on peptide integrity and biological activity should be further investigated. Future studies should optimize particle size distribution and preparation processes. Continuous production technologies should also be explored to support practical application in functional food systems. In addition, *in vivo* animal studies are needed to systematically evaluate bioavailability and biological activity. The application of this delivery system to other marine bioactive peptides should also be further investigated.

## Figures and Tables

**Table 1:** Factors and levels of experiment.

Level	Factor		
	A: SA (%, *w*/*v*)	B: CS (%, *w*/*v*)	C: CaCl_2_ (%, *w*/*v*)
−1	1.00	0.50	1.50
0	1.50	1.00	2.00
1	2.00	1.50	2.50

**Table 2:** Design of RSM experiments and EE of SCPs.

Run	A: SA (%, *w*/*v*)	B: CS (%, *w*/*v*)	C: CaCl_2_ (%, *w*/*v*)	EE (%)
1	0	−1	−1	83.96
2	0	0	0	93.20
3	0	0	0	92.78
4	0	0	0	93.11
5	0	−1	1	89.16
6	−1	0	−1	83.21
7	0	1	−1	81.54
8	1	−1	0	89.77
9	1	1	0	90.07
10	1	0	1	89.98
11	−1	0	1	88.92
12	−1	1	0	87.09
13	1	0	−1	84.82
14	0	0	0	93.09
15	0	0	0	92.91
16	0	1	1	89.06
17	−1	−1	0	90.09

**Table 3:** ANOVA for response surface regression model.

Source	Sum of Squares	df	Mean Square	F-Value	*p*-Value	Significant
Model	219.91	9	24.43	312.74	<0.0001	**
A	3.55	1	3.55	45.45	0.0003	**
B	3.41	1	3.41	43.59	0.0003	**
C	69.56	1	69.56	890.25	<0.0001	**
AB	2.72	1	2.72	34.84	0.0006	**
AC	0.0756	1	0.0756	0.9679	0.3580	-
BC	1.35	1	1.35	17.22	0.0043	**
A^2^	9.23	1	9.23	118.07	<0.0001	**
B^2^	21.94	1	21.94	280.80	<0.0001	**
C^2^	97.22	1	97.22	1244.27	<0.0001	**
Residual	0.5470	7	0.0781			
Lack of Fit	0.4319	3	0.1440	5.00	0.0769	-
Pure Error	0.1151	4	0.0288			
Cor Total	220.45	16				
R^2^	0.9975					
R^2^_Adj_	0.9943					

** Highly significant effect (*p* < 0.01). - Insignificant effect (*p* > 0.05).

**Table 4:** Average particle size, EE, and LC of gel beads.

Samples	Average Particle Size (mm)	EE (%)	LC (g/g)
SCP-SM	1.41 ± 0.14	58.61 ± 0.92	27.04 ± 1.23
SCP-BM	1.95 ± 0.11	94.33 ± 0.46	31.91 ± 0.79

## Data Availability

The original contributions presented in the study are included in the article, further inquiries can be directed to the corresponding author.

## Abbreviations

The following abbreviations are used in this manuscript:

SCP	Sea cucumber mouthpart peptide
SA	Sodium alginate
CS	Chitosan
SCP-BM	Double-layer gel bead
SCP-SM	Single-layer gel bead
BM	Blank double-layer gel bead
SM	Blank single-layer gel bead
EE	Encapsulation efficiency
